# Automatic, Qualitative Scoring of the Interlocking Pentagon Drawing Test (PDT) Based on U-Net and Mobile Sensor Data

**DOI:** 10.3390/s20051283

**Published:** 2020-02-27

**Authors:** Ingyu Park, Yun Joong Kim, Yeo Jin Kim, Unjoo Lee

**Affiliations:** 1 Department of Electronic Engineering, Hallym University, Chuncheon 24252, Korea; qkrdlsrb3946@naver.com; 2 Department of Neurology, Hallym University Sacred Heart Hospital, Hallym University College of Medicine, Hallym University, Anyang 14068, Korea; yunkim@hallym.ac.kr; 3 Department of Neurology, Chuncheon Sacred Heart hospital, Hallym University College of Medicine, Chuncheon 24252, Korea

**Keywords:** pentagon drawing test, automatic scoring, mobile sensor, deep learning, U-Net, Parkinson’s disease

## Abstract

We implemented a mobile phone application of the pentagon drawing test (PDT), called mPDT, with a novel, automatic, and qualitative scoring method for the application based on U-Net (a convolutional network for biomedical image segmentation) coupled with mobile sensor data obtained with the mPDT. For the scoring protocol, the U-Net was trained with 199 PDT hand-drawn images of 512 × 512 resolution obtained via the mPDT in order to generate a trained model, Deep5, for segmenting a drawn right or left pentagon. The U-Net was also trained with 199 images of 512 × 512 resolution to attain the trained model, DeepLock, for segmenting an interlocking figure. Here, the epochs were iterated until the accuracy was greater than 98% and saturated. The mobile senor data primarily consisted of x and y coordinates, timestamps, and touch-events of all the samples with a 20 ms sampling period. The velocities were then calculated using the primary sensor data. With Deep5, DeepLock, and the sensor data, four parameters were extracted. These included the number of angles (0–4 points), distance/intersection between the two drawn figures (0–4 points), closure/opening of the drawn figure contours (0–2 points), and tremors detected (0–1 points). The parameters gave a scaling of 11 points in total. The performance evaluation for the mPDT included 230 images from subjects and their associated sensor data. The results of the performance test indicated, respectively, a sensitivity, specificity, accuracy, and precision of 97.53%, 92.62%, 94.35%, and 87.78% for the number of angles parameter; 93.10%, 97.90%, 96.09%, and 96.43% for the distance/intersection parameter; 94.03%, 90.63%, 92.61%, and 93.33% for the closure/opening parameter; and 100.00%, 100.00%, 100.00%, and 100.00% for the detected tremor parameter. These results suggest that the mPDT is very robust in differentiating dementia disease subtypes and is able to contribute to clinical practice and field studies.

## 1. Introduction

The pentagon drawing test (PDT) is a sub-test of the Mini-Mental State Examination (MMSE), used extensively in clinical and research settings as a measure of cognitive impairment [1]. The MMSE is a general screening tool for cognitive impairment. However it shows low sensitivity for detecting cognitive impairment in Parkinson’s disease [2]. 

Parkinson’s disease is the second most common neurodegenerative disease and is characterized by motor symptoms [3]. In addition, 83% of long-term survivors of Parkinson’s disease showed dementia, with impairment of their visuospatial functions and executive functions [4]. As up to half of Parkinson’s patients show visuospatial dysfunction, the PDT has also been used for the distinction of dementia in Parkinson’s disease and Alzheimer’s disease cases [5,6,7,8,9,10]. However, the conventional PDT scoring method shows a low sensitivity in detecting visuospatial dysfunction in Parkinson’s disease. It is necessary to improve the scoring method of the PDT to measure visuospatial dysfunction more accurately.

Visuospatial functioning includes various neurocognitive abilities, such as coordinating fine motor skills, identifying visual and spatial relationship among objects, and planning and executing tasks [9]. These functions allow us to estimate distance and perceive depth, use mental imagery, copy drawings, and construct objects or shapes [11]. Before clinical symptoms of Parkinson’s or Alzheimer’s disease become manifest, various visuospatial dysfunctions are known to be detectable for several years before, thus indicating that visual spatial assessment can assist in making an early and accurate diagnosis of these cases [12]. 

There are several tests including the clock drawing test (CDT) and the Rey–Osterrieth complex figure (ROCF) test, in addition to the PDT, for measuring visuospatial function. The CDT is applied for early screening of cognitive impairment, especially in dementia. It can be used to assess tasks, such as following directions, comprehending language, visualizing the proper orientation of an object, and executing normal movements. These may be disturbed in dementia [13,14]. Due to the dominant roles of the CDT in dementia, different types of scoring systems are relatively well established, ranging from semi-quantitative to qualitative scoring systems [15,16]. There are also several suggested methods for performing the CDT in a digital environment and/or scoring it based on machine learning, convolutional neural networks, and ontology representations [17,18,19,20]. 

The ROCF test employs a complex geometrical figure for the stimulus, comprised of a large rectangle with horizontal and vertical bisectors, two diagonals, and additional geometric details [21]. The ROCF is widely used for testing visual perception and long-term visual memory with its usefulness in providing information on the location and extent of any damage, and through the order and accuracy in which the figure is copied and drawn from recall [22]. Various scoring systems for the ROCF test, including the Osterrieth system and the Boston Qualitative scoring system, have been employed to derive a more quantitative value for the accuracy of a subject’s drawing. However, these quantify the accuracy of the drawings by hand and in a subjective manner due to the complexity of the figures [23]. To address this, an automated scoring algorithm for the ROCF test was recently suggested and is based on cascaded deep neural networks, trained on scores from human expert raters [24].

As for the PDT, it is a relatively fast, simple, and sensitive test compared to the CDT and the ROCF tests. For the test, a subject is asked to copy or draw two interlocking pentagons with the interlocking shape being a rhombus. The interpretation of PDT is usually binary, but there are several ways of interpreting PDT such as a feasible scoring for a total of 6 or 10 points [25,26], a standardized scoring for use in psychiatric populations [27], and a qualitative measure with a total of 13 points [5]. The established scores for a total of 6 points are as follows: 6 points for correct copying; 5 points for two overlapping pictures, one being a pentagon; 4 points for only two overlapping pictures; 3 points for having two figures, not overlapping; 2 points for a closed figure; and 1 point when the drawing does not have the shape of a closed figure. The differences in the length of the sides were additionally considered in the scoring, giving an overall total of 10 points. The standardized scoring increased the sensitivity and reliability for use in nonorganic psychiatric patients by also qualitatively considering the distortion degree of the pentagon shapes. The qualitative scoring involved the assessment of drawing parameters, including the number of angles, distance/intersection, closure/opening, rotation, and closing-in, in the hand-drawn images for the PDT. 

Many studies have shown evidence for the PDT being prognostic for the assessment of visuospatial functions. The PDT has been used in the differentiation of dementia with Lewy Bodies, Alzheimer’s disease, Parkinson’s disease (with or without dementia), paraphrenia, schizophrenia, and obsessive compulsive disorder, with the qualitative scoring systems applied rather than using the binary scoring system [6,9,26,27,28,29,30]. An associative study of the PDT and the CDT has also shown the PDT to be applicable as a prognostic marker in dementia with Lewy Bodies [31]. From these studies, the qualitative scoring of the PDT is practical and effective in distinguishing several clinical features of various cognitive deficits. However, as the qualitative scoring of the PDT is done manually, it can be prone to human error and is not very practical in analyzing big data, such as for personal lifelogs. With the PDT result being qualitative and not numeric, the results are also difficult to evaluate objectively. As such, the necessity of a standardized, qualitative, and automatic scoring system for the PDT has increased. The availability of digital and mobile sensors, coupled with deep learning algorithms, has made it possible to think of ways to using this technology to obtain information for the PDT and to add order and accuracy for interpreting PDT figures as they are being copied and drawn from recall. This may provide additional information on the location and extent of any damage.

In this study, we developed a mobile-phone application version of the PDT, named the mPDT, and a novel automatic, qualitative scoring system, based on U-Net, a convolutional network for biomedical image segmentation (see Appendix A for the summary and comparison of this study to those of other reports). The mobile sensor data from a smart phone allowed for development of this mPDT application and the scoring system. The U-Net was trained with 199 PDT hand-drawn images of 512 × 512 resolution obtained via mPDT to generate a trained model, Deep5, in segmenting the drawn right and left pentagon images. The U-Net was also trained with the same 199 PDT 512 × 512 resolution images for DeepLock, also a trained model, for segmenting an interlocking image of two pentagons. 

**Figure 1:**
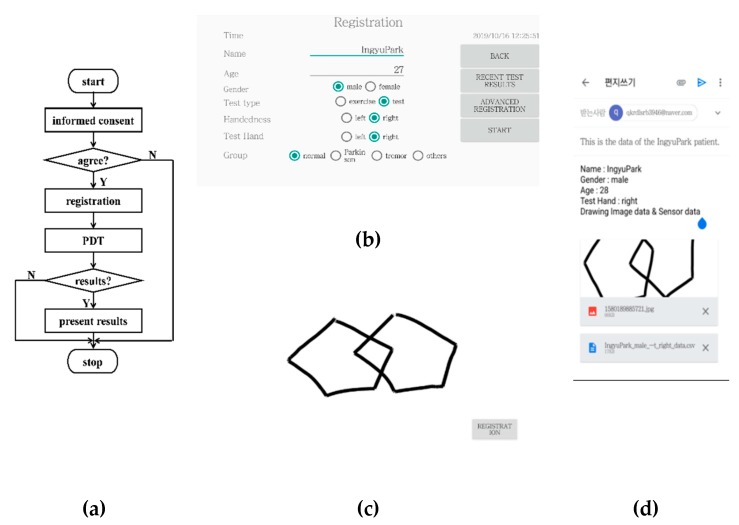
(**a**) Flow diagram of the mobile phone pentagon drawing test (PDT) (mPDT) operation; screen shots of the (**b**) registration window; (**c**) PDT window; and (**d**) results window of the mPDT.

**Figure 2:**
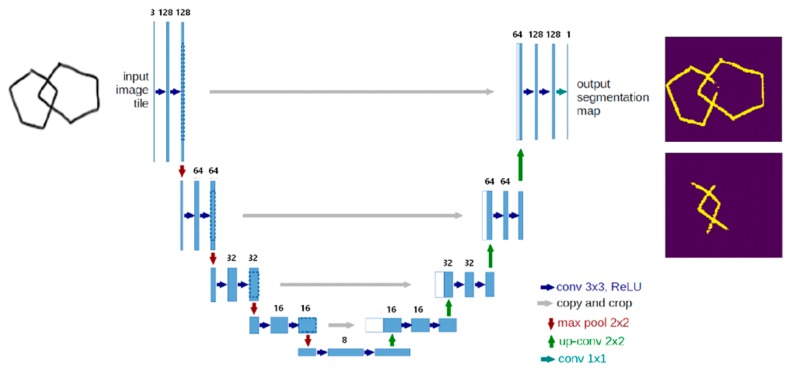
The U-Net network architecture used in this study.

**Figure 3:**
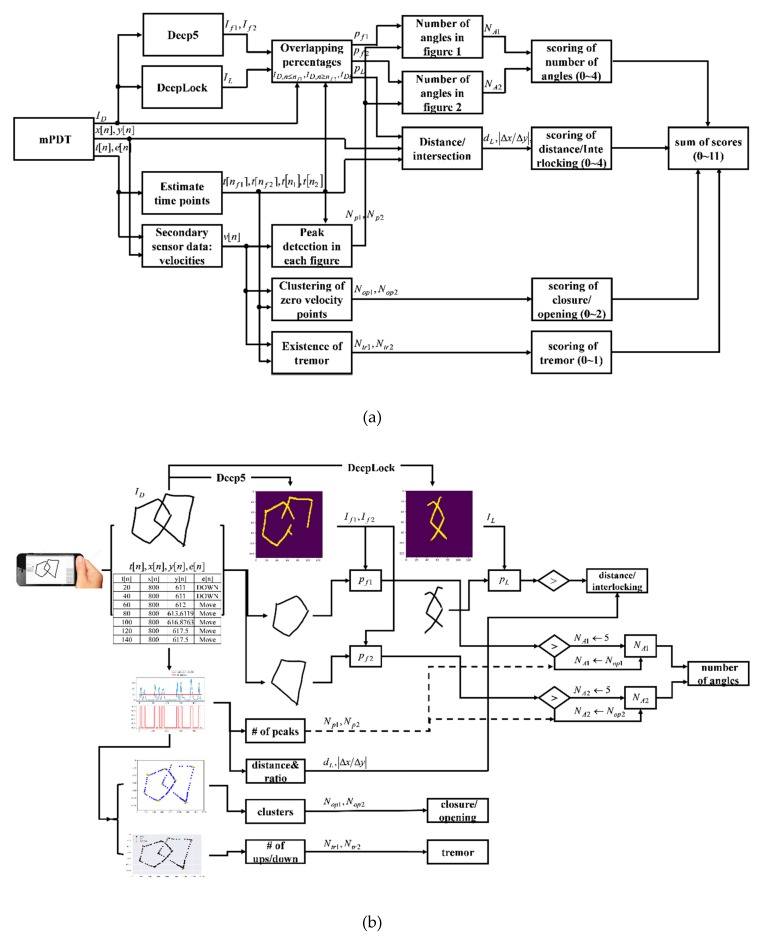
(**a**) Overall flowchart of the scoring method; (**b**) Schematic diagram of the scoring method.

**Figure 4:**
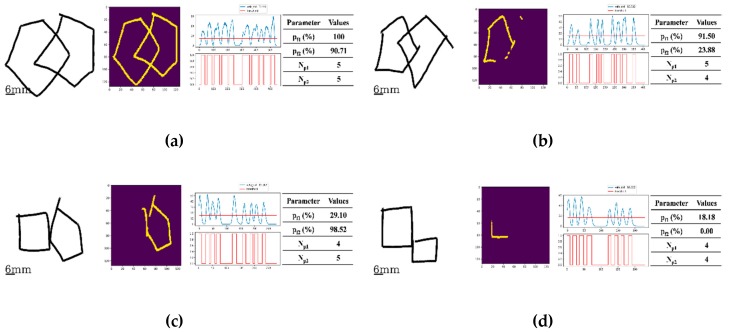
Four examples of original drawings (left) along with their segmented images (middle left) produced by the pre-trained model, Deep5, their corresponding velocity graphs (middle right), and the corresponding parameter values (right). The number of angles was evaluated from the number of peaks detected in the velocity graph: (**a**) The image is composed of two interlocking figures, both being pentagons where the number of angles for both figures was evaluated to be 5 ($p_{f1}$ and $p_{f2}$ were 100.00% and 90.71%, respectively, and both greater than 75%). (**b**) The image of two interlocking figures, but with only one being a pentagon (the left-side figure). The number of angles of the left-side figure was evaluated to be 5, as $p_{f1}$ was 91.5% and it was greater than 75%. For the right-side figure, the number of angles was given as 4, as the percentage $p_{f2}$ of 23.88% was less than 75% and the number of peaks detected in the velocity graph is 4. (**c**) The image of the right portion is a pentagon with the number of angles evaluated as 5 as $p_{f2}$ was 98.52% and it was greater than 75%. The image of the left portion was evaluated as a 4, as $p_{f1}$ was 29.10% and it was less than 75% and the number of peaks detected in the velocity graph is 4. (**d**) The image, composite of two figures, none of them being a pentagon. This is because the number of angles for each of the right and left-hand portions was evaluated to be a 4, since $p_{f1}$ and $p_{f2}$ were 18.18% and 0.00%, respectively, both being less than 75%. The numbers of angles in both the right and left portions were evaluated to be 4, matching the number of peaks detected in the velocity graphs for both.

The epochs were then iterated for both Deep5 and DeepLock until the accuracies were greater than 98% and saturated as well. The mobile sensor data consisted of primary and secondary data, where the primary data were the x and y coordinates, timestamps, and touch events for all the samples with a 20 ms sampling period extracted from the mobile touch sensor. The secondary data were the velocities calculated using the primary data. Four parameters, including the number of angles (0–4 points), distance/intersection between the two figures (0–4 points), closure/opening of the contour (0–2 points), and detected tremors (0–1 points), were estimated using Deep5 and DeepLock and the sensor data, resulting in scaling with a total of 11 points. 

The performance test was performed with images and sensor data from 230 subjects, obtained via the mPDT. All the images were scored by two clinical experts in PDT scaling for an objective performance analysis. The results of the performance test indicated the sensitivity, specificity, accuracy, and precision for the number of angles at 97.53%, 92.62%, 94.35%, and 87.78%; for the distance/intersection at 93.10%, 97.90%, 96.09%, and 96.43%; for the closure/opening at 94.03%, 90.63%, 92.61%, and 93.33%; and for tremors at 100.00%, 100.00%, 100.00%, and 100.00%, respectively. These results suggest that our mPDT application is valuable in differentiating dementia disease subtypes and also useful for clinical practice and field studies. 

## 2. Materials and Methods

### 2.1. Subjects

The Institutional Review Board of the Hallym University Sacred Heart Hospital approved this study. A total of 328 right-handed young volunteers (175 males and 153 females, aged (mean ± std.) 23.98 ± 2.83 years) were recruited and participated in the pentagon drawing test using the mPDT, our mobile application developed in this study. In addition, 101 pentagon drawing images by Parkinson’s disease (PD) patients (47 males and 54 females, aged (mean ± std.) 75.86 ± 8.13 years) that visited the university hospital were used in this study. The pentagon drawing image data of 199 volunteers (107 males and 92 females, aged (mean ± std.) 22.11 ± 1.44 years) from the aforementioned 328 volunteers were used in creating the pre-training models of Deep5 and DeepLock, using the U-Net algorithm. The remaining 129 volunteers (68 males and 61 females, aged (mean ± std.) 26.87 ± 1.84 years) provided the pentagon drawing image data, along with those from the 101 PD patients, that were used in testing the scoring method. All the images were scored by two clinical experts in PDT scaling. Table 1 summarizes certain informative statistics of age, gender, and binary PDT score of the 328 volunteers and 101 PD patients.

### 2.2. Implementation of the Mobile Pentagon Drawing Test, mPDT

The mobile application, mPDT, for the interlocking pentagon test was developed using the Android Studio development environment. While the source code of mPDT was implemented to be able to be built in any mobile device, including smartphones, tablets, or notebooks, we built and tested the mPDT in a Samsung Galaxy Note 4 smartphone with a resolution of 640 dots per inch and a spatial accuracy of 0.004 cm. The mPDT allows for a user to copy two interlocking pentagons (with the interlocking shape being a rhombus) on the screen, and scores the drawing image qualitatively based on the sensor data of the drawing image and the pre-trained models, Deep5 and DeepLock, developed in this study. Using U-Net, a convolutional network architecture for fast and precise segmentation of images, the pre-trained models Deep5 and DeepLock were generated for segmenting the pentagon shapes and the interlocking shape, respectively. 

The sensor data collected by the touch sensors embedded in a smartphone consisted of timestamps in seconds, the x and y coordinates in pixels, and touch-events of the samples of the drawing image with a 50 Hz sampling frequency. Figure 1a shows the flow diagram of the mPDT operation in, and Figure 1b–d shows the screen shots of the registration window, the PDT window, and the result window for mPDT, respectively. At the launch of mPDT, an informed consent prompt appears, following this, a registration window is displayed, where it is possible to enter the subject’s information, such as name, age, gender, and handedness, plus the optional parameters. 

Following the user pressing the start button in the registration window, the PDT window then appears, in which the user is asked to copy two interlocking pentagons provided on a paper by an examiner or draw them while recalling from an image provided on a previous window. Values of measured parameters for the time in seconds and the x and y coordinates in pixels are obtained from the drawing image with a 50 Hz sampling frequency. The values are saved as sensor data when the subject copies or draws pentagons on the touch screen of the PDT window. The results window then displays the sensor data along with the drawn image, and/or a plot of speeds in mm/sec of inter-samples over time. The results could then be sent to the email address entered at the registration window.

### 2.3. Pre-Trained Models, Deep5 and DeepLock based on the U-Net 

Novel pre-trained models of Deep5 and DeepLock were developed for segmentation of the drawn pentagon portions and the interlocking domains of the images, respectively. Deep5 and DeepLock were created based on the U-Net convolutional network architecture in keras [32]. The network architecture implemented in this study is illustrated in Figure 2. It consists of a contracting path, an expansive path, and a final layer. The contracting path consists of repeated applications of two 3 × 3 convolutions and a 2 × 2 max pooling operation with stride 2 for down-sampling. At each repetition, the number of feature channels is doubled. The expansive path consists of two 3 × 3 convolutions and a 2 × 2 convolution (“up-convolution”) for up-sampling to recover the size of the segmentation map. At the final layer, a 1 × 1 convolution was used to map each 16-component feature vector to the desired number of classes. In total, the network has 23 convolutional layers. The training data for both Deep5 and DeepLock contain 960 images of 128 × 128 resolution, which were augmented using a module called ImageDataGenerator in keras.preprocessing.image and resized from the original 199 images of 1600 × 1320 resolution. Deep5 and DeepLock were generated by training the network architecture for five and seven epochs with accuracies of approximately 0.977 and 0.979, respectively. The loss function used for the training was essentially binary cross entropy. 

### 2.4. Scoring Method of mPDT

The novel, automatic, and qualitative scoring method for the mPDT was developed based on the sensor data and the pre-trained models, Deep5 and DeepLock. Four parameters were included in the scoring method: the number of angles (0–4 points), distance/intersection between the two figures (0–4 points), closure/opening of the image contour (0–2 points), and detected tremors (0–1 points). All the assigned scores for the parameters are integers. A total score corresponding to the sum of individual scores of each parameter ranged from 0 to 11. The parameters, number of angles (0–4 points), distance/intersection between the two figures (0–4 points), and closure/opening of the image contour (0–2 points) were adopted from a previous study by Paolo Caffarra et al. [5]. When a subject executes more than one copy of the pentagons, the last copy is then scored. A detailed list of the parameters used is presented in Table 2 and the overall flowchart and the schematic diagram of the scoring method are shown in Figure 3a,b, respectively. 

The scoring method consists of a series of processes that include manipulation of the sensor data and segmentation of the drawn pentagon and the interlocking shapes using Deep5 and DeepLock, respectively. There is then the extraction of variables (the number of angles, distance/intersection, closure/opening, and presence of tremors) and the assignment of scores according to the performance scores for each parameter. The sensor data primary obtained from the drawn image samples during the mPDT interaction include timestamps, x- and y-coordinates, and touch events. The time index of moving from a figure drawing to another is detected from the primary sensor data in the process of the manipulation of the sensor data. Velocity values are also obtained from the primary sensor data in the process of the manipulation of sensor data. 

The drawn image is then analyzed by Deep5 and DeepLock to respectively segment the pentagon and the interlocking shapes. It is here that the percentages of the segmented pentagon shapes and the interlocking shape overlapping the drawn image are calculated. Next, the values for each parameter are calculated. For the total number of angles of each figure, the percentages and the number of peaks in velocities are determined. For the distance/intersection, the interlocking distance between two figures and the absolute ratio of the differences in x and y coordinates of the two points used in the calculation of the interlocking distance are extracted. After clustering the primary sensor data of zero velocities, the mean distance between the cluster center and the points belonging to each cluster are calculated for the closure/opening parameter. For presence of tremors, the frequency of consecutive ‘up and down’ touch events from the sensor data is estimated. Finally, there is the assignment of integer scores according to each parameter score, as in Table 2 (also see Appendix B for the examples of drawn images and the corresponding performance scores and assigned integer scores). The details are described in the following subsections.

#### 2.4.1. Sensor Data Manipulation and Shape Segmentation Using Deep5 and Deeplock

Once the mPDT has been completed, three pieces of data, including the drawing image and the primary and secondary sensor data, are generated for output. The size of the drawing image $I_{D}$ is 128 × 128 pixels, which is resized from the original 1600 × 1320 drawing image of the PDT window. The primary sensor data consist of times t[n] in seconds, x- and y- coordinates, x[n] and y[n] in pixels, and touch-events e[n] of the sample points of the 128 × 128 drawing image, where the sampling rate was set at 50 *Hz* and n is the index of a sample point. The secondary sensor data are velocities v[n] in pixels/sec which are calculated from the primary sensor data. Each of the touch-events e[n] has one of the values, such as -1, 0, 1, where the assigned value of -1 is for the event ‘down’, as in touching on the screen; 1 for the event ‘up’, as in touching off the screen; and 0 for the event ‘move’, as in moving and touching on the screen. For the drawing image $I_{D}$, it is supposed to be of two interlocking pentagons. 

The sensor data for each of the two interlocking shapes is separately obtained using the times $t[n_{f1}]$ of the last sample point of the first drawn figure and $t[n_{f2}]$ of the first sample point of the second drawn figure. In other words, the sample points for t[n], $n\leq n_{f1}$ belong to the first figure $I_{D,n\leq n_{f1}}$ and those for t[n], $n\geq n_{f2}$ to the second figure $I_{D,n\geq n_{f2}}$. The time $t[n_{f1}]$ can be estimated by the touch-events shifting from the event ‘move’ into the event ‘up’ and then successively staying at the event ‘up’ for the longest time if such a period occurs more than once. Therefore, the index $n_{f1}$ can be determined by finding the longest chain of events consisting of a 0 (the event ‘move’) and consecutive 1s (the ‘up’ events) in the sequence of touch-events e[n]. The time $t[n_{f2}]$ can be obtained by adding the longest period of time to the time $t[n_{f1}]$. Sensor data for the interlocking image $I_{DL}$ of the 128 × 128 drawing image $I_{D}$ is obtained from the sample points between two data points, $\left(x[n_{1}],y[n_{1}]\right)$ and $\left(x[n_{2}],y[n_{2}]\right)$, where $n_{1}$ is the index at which the value of $x[n_{1}]$ is the maximum of those belonging to x[n] ($n<n_{f1}$) and $n_{2}$ is the index at which the value of $x[n_{2}]$ is the minimum of those belonging to x[n] ($n\geq n_{f2}$).

The two pentagon shapes $I_{fi}$(i=1,2) are then separately segmented using indices $n_{fi}$(i=1,2) and the Deep5 pre-trained model from the 128 × 128 drawing image $I_{D}$. An interlocking shape $I_{L}$ is segmented using the DeepLock pre-trained model from the 128 × 128 drawing image $I_{D}$ as well. Next, percentage $p_{fi}$(i=1,2) of the segmented image $I_{fi}$(i=1,2) matching to the corresponding figure of the 128 × 128 drawing image $I_{D}$ is estimated as below:

$$p_{f1}=\frac{n(I_{D,n\leq n_{f1}}\cap I_{f1})}{n(I_{D,n\leq n_{f1}})},\mathrm{and}$$

$$p_{f2}=\frac{n(I_{D,n\geq n_{f2}}\cap I_{f2})}{n(I_{D,n\geq n_{f2}})},$$

where $n(I_{D,n\leq n_{f1}}\cap I_{f1})$ is the number of pixel coordinates that *I_f_*_1_ and the first figure of the 128 × 128 drawing image $I_{D}$ have in common; $n(I_{D,n\leq n_{f1}})$ is the total number of pixel coordinates in the first figure of the 128 × 128 drawing image $I_{D}$. Similarly, $n(I_{D,n\geq n_{f2}}\cap I_{f2})$ is the number of pixel coordinates that $I_{f2}$ and the second figure of the 128 × 128 drawing image $I_{D}$ have in common; $n(I_{D,n\geq n_{f2}})$ is the total number of pixel coordinates in the second figure of the 128 × 128 drawing image $I_{D}$. The percentage $p_{L}$ of the image $I_{L}$ matching to the interlocking image $I_{DL}$ of the 128 × 128 drawing image $I_{D}$ is estimated as follows:

$$p_{L}=\frac{n(I_{DL}\cap I_{L})}{n(I_{DL})},$$

where $n(I_{DL}\cap I_{L})$ is the number of pixel coordinates that $I_{L}$ and $I_{DL}$ have in common; $n(I_{DL})$ is the total number of pixel coordinates in $I_{DL}$.

#### 2.4.2. Number of Angles

Number of angles is the sum of the number $N_{Ai}$ (i=1,2) of angles of each figure of the 128 × 128 drawing image $I_{D}$, which is estimated using the percentage, $p_{fi}$ (i=1,2), velocity v[n], and the index $n_{fi}$(i=1,2) as follows:

$$N_{Ai}=\{\begin{array}{cc} 5 & p_{fi}>p_{th1}\\ N_{pi} & else \end{array},$$

where $N_{pi}$ (i=1,2) is the number of peaks in velocity v[n] for each figure. If the percentage $p_{fi}$(i=1,2) of the segmented image $I_{fi}$(i=1,2) matching to the corresponding figure of the 128 × 128 drawing image $I_{D}$ is larger than a given threshold $p_{th1}$, then the shape of the figure is identified as a pentagon and the number $N_{Ai}$ (i=1,2) of angles is estimated to be 5. If not, then the number $N_{A1}$ and $N_{A2}$ are estimated by the numbers $N_{p1}$ and $N_{p2}$ of peaks in velocity v[n] ($n\leq n_{f1}$) and v[n] ($n\geq n_{f2}$), respectively, since the velocity of the drawing stroke increases and then decreases to zero at the point of change in direction.

#### 2.4.3. Distance/Intersection between Two Figures

The distance/intersection between two figures is evaluated by the percentage $p_{L}$ of the image $I_{L}$ matching to the interlocking image $I_{DL}$ of the 128 × 128 drawing image $I_{D}$, the distance $d_{L}$ between two figures in the interlocking image, and the ratio Δx/Δy of differences of two data points $\left(x[n_{1}],y[n_{1}]\right)$ and $\left(x[n_{2}],y[n_{2}]\right)$. The distance $d_{L}$ in cm between two figures in the interlocking image is estimated by the distance between two data points, $\left(x[n_{1}],y[n_{1}]\right)$ and $\left(x[n_{2}],y[n_{2}]\right)$, where $n_{1}$ is the index at which the value of $x[n_{1}]$ is the maximum of those belonging to x[n] ($n\leq n_{f1}$) and $n_{2}$ is the index at which the value of $x[n_{2}]$ is the minimum of those belonging to x[n] ($n\geq n_{f2}$). The ratio Δx/Δy is estimated by the ratio of the differences Δx and Δy, where Δx and Δy are the differences in cm in x- and y-axis directions, respectively, between two data points, $\left(x[n_{1}],y[n_{1}]\right)$ and $\left(x[n_{2}],y[n_{2}]\right)$. The existence and the shape of the interlocking between two figures can be discriminated by the percentage $p_{L}$ of the segmented interlocking image $I_{L}$ matching to the interlocking image $I_{DL}$ of the 128 × 128 drawing image $I_{D}$, the distance $d_{L}$, and the ratio Δx/Δy. 

If the percentage $p_{L}$ is larger than a given threshold $p_{th2}$, the distance $d_{L}$ has a negative value and the absolute value of the ratio |Δx/Δy| is larger than a given threshold $r_{th}$, the two figures are then evaluated to be interlocked with a shape of a rhombus. If the percentage $p_{L}$ is larger than a threshold $p_{th2}$ and the distance $d_{L}$ has a negative value but the absolute value of the ratio |Δx/Δy| is less than the given threshold $r_{th}$, then the two figures are evaluated to be interlocked without the shape of a rhombus. On the other hand, if the distance *d_L_* has a positive value regardless of the percentage $p_{L}$ and the absolute value of the ratio |Δx/Δy|, then the two figures are evaluated as not to be interlocked and apart from each other by the distance $d_{L}$. In such a case, if the value of the distance $d_{L}$ is nearly positively zero, being less than ε, a very small positive value, then the two figures are evaluated as to be attached to each other. The evaluation of the distance $d_{L}$ and the absolute ratio |Δx/Δy| are formulated as follows:

$$d_{L}=\{\begin{array}{cc} -\sqrt{{\left(x\left[n_{1}\right]-x\left[n_{2}\right]\right)}^{2}+{\left(y\left[n_{1}\right]-y\left[n_{2}\right]\right)}^{2}}, & x\left[n_{1}\right]>x\left[n_{2}\right]\\ \sqrt{{\left(x\left[n_{1}\right]-x\left[n_{2}\right]\right)}^{2}+{\left(y\left[n_{1}\right]-y\left[n_{2}\right]\right)}^{2}} & else \end{array},\mathrm{and}$$

$$\left|\Delta x/\Delta y\right|=|\frac{x\left[n_{1}\right]-x\left[n_{2}\right]}{y\left[n_{1}\right]-y\left[n_{1}\right]}|.$$

#### 2.4.4. Existence of Closure/Opening

Existence of opening in a figure is determined by the fact that an opening could exist in a region with a relatively larger variance of distances between sample points, having consecutive 0s of velocity in a figure, as there are changes in the stroke direction as well as in the stroke position within the region where an opening occurs. Parameters of the existence of openings $N_{op1}^{}$ and $N_{op2}^{}$ in the figure images of $I_{D,n\leq n_{f1}}$ and $I_{D,n\geq n_{f2}}$ are estimated by k-means clustering of the sample points (x[n],y[n]), $n\leq n_{f1}$ and (x[n],y[n]), $n\geq n_{f2}$ with zero velocity, respectively, where the target numbers for the parameters $N_{op1}^{}$ and $N_{op2}^{}$ are set to the numbers of angles $N_{A1}$ and $N_{A2}$, respectively. Then, the cluster parameter $k_{ij}$ ($i=1,2,j=1,\ldots N_{Ai}$) is calculated by averaging the distances between the sample points belonging to the cluster $\delta_{ij}$ and the cluster center $\delta_{c,ij}$ as follows:

$$k_{ij}=mean\left({\left|(x\left[n\right],y\left[n\right])-\delta_{c,ij}\right|}_{(x\left[n\right],y\left[n\right])\in \delta_{ij}}\right).$$

The parameters of the existence of openings $N_{op1}^{}$ and $N_{op2}^{}$ in the figure images of $I_{D,n\leq n_{f1}}$ and $I_{D,n\geq n_{f2}}$ are then estimated as follows:

$$N_{opi}=\{\begin{array}{cc} 1, & \begin{array}{ccccc} \left(k_{i1}>\delta_{th}\right) & or & \cdots & or & \left(k_{iN_{Ai}}>\delta_{th}\right) \end{array}\\ 0, & else \end{array},$$

where $\delta_{th}$ is a given threshold.

#### 2.4.5. Existence of Tremors

A tremor is an involuntary quivering movement or shake which could be caused by age-associated weakness, neurodegenerative diseases, or mental health conditions. A tremor can be detected from the frequency of a ‘down’ event happening after an ‘up’ event in the chain of touch-events, which cannot be observed in a paper and pencil test. The existence of tremors $N_{tri}$ (i=1,2) in each figure is determined by the total number of ‘up’ events followed by ‘down’ events being larger than a given threshold $e_{th}$ in the touch-events e[n] of the sample points in the corresponding figure. An ‘up’ event followed by a ‘down’ event in the touch-events e[n] can be detected when the multiplication of two neighboring values in touch-events e[n] is equal to -1 as the values of ‘up’ and ‘down’ events are set to 1 and -1, respectively. The number of tremors $N_{tri}$ (i=1,2) in each figure is evaluated as follows:

$$N_{tr1}=\{\begin{array}{cc} 1, & \sum_{n\leq n_{f1}}e[n]e[n+1]\geq e_{th}\\ 0, & else \end{array},\mathrm{and}$$

$$N_{tr2}=\{\begin{array}{cc} 1, & \sum_{n\geq n_{f2}}e[n]e[n+1\geq e_{th}\\ 0, & else \end{array}$$.

#### 2.4.6. Assignment of Scores

Table 3 lists the conditions for the assigned integer scores for each parameter in the mPDT. The score of the number of angles is via percentages, $p_{f1}$ and $p_{f2}$, in addition to the numbers of angles, $N_{A}{}_{1}$ and $N_{A}{}_{2}$. The score is a 4 if both of the percentages, $p_{f1}$ and $p_{f2}$, are equal or greater than a given threshold $p_{th1}$; the score is a 3 if at least one of the percentages, $p_{f1}$ or $p_{f2}$, is less than $p_{th1}$ as well as the sum number of angles, $N_{A}{}_{1}+N_{A2}$, is 9 or 11; the score is a 2 if at least one of the percentages, $p_{f1}$ or $p_{f2}$, is less than $p_{th1}$ as well as the sum number of angles, $N_{A}{}_{1}+N_{A2}$, is 8 or 12; the score is a 1 if at least one of the percentages, $p_{f1}$ or $p_{f2}$, is less than $p_{th1}$ as well as the sum number of angles, $N_{A}{}_{1}+N_{A2}$, is between or including 5 and 7; and the score is a 0 if at least one of the percentages, $p_{f1}$ or $p_{f2}$, is less than $p_{th1}$ as well as the sum number of angles, $N_{A}{}_{1}+N_{A2}$, is less than 5 or greater than 12.

The score for distance/intersection is obtained by using the percentage *p_L_*, the distance $d_{L}$ between the two figures in the interlocking image, and the absolute value of the ratio |Δx/Δy|. The score is a 4 if the percentage $p_{L}$ is equal to or larger than a given threshold $p_{th2}$, the distance $d_{L}$ is less than −ε, and the absolute value of the ratio |Δx/Δy| is equal to or larger than a given threshold $r_{th}$; the score is a 3 if the percentage $p_{L}$ is less than $p_{th2}$, the distance $d_{L}$ is less than −ε, and the absolute value of the ratio |Δx/Δy| is less than a given threshold $r_{th}$; the score is a 2 if the percentage $p_{L}$ is less than $p_{th2}$ as well as the absolute of the distance $d_{L}$ is equal to and less than ε; the score is a 1 if the percentage $p_{L}$ is less than $p_{th2}$ as well as the distance $d_{L}$ is between ε and 1 cm exclusive; and the score is a 0 if the percentage $p_{L}$ is less than $p_{th2}$ as well as the distance $d_{L}$ is equal to or greater than 1 cm.

The score of closure/opening is determined by the parameters $N_{op1}^{}$ and $N_{op2}^{}$. The score is a 2 if there are no openings in both figures ($N_{op1}^{}$$N_{op2}^{}$ = 1 and $N_{op1}^{}$+$N_{op2}^{}$ = 2); the score is a 1 if there is an opening in one of the two figures ($N_{op1}^{}$$N_{op2}^{}$= 0 and $N_{op1}^{}$+$N_{op2}^{}$ = 1); and the score is a 0 if there are openings in both figures ( $N_{op1}^{}$$N_{op2}^{}$ = 0 and $N_{op1}^{}$+$N_{op2}^{}$ = 0).

The score for tremors is determined by the values $N_{tr1}$ and $N_{tr2}$. It is given a score of 1 if both $N_{tr1}$ and $N_{tr2}$ are equal to 1 ($N_{tr1}^{}N_{tr2}=1$); and 0 if any of $N_{tr1}$ and $N_{tr2}$ values is equal to 0 ($N_{tr1}^{}N_{tr2}=0$). 

## 3. Results

### 3.1. Scoring of the Number of Angles

Figure 4 depicts separate examples of original drawings, each with their own characteristic shapes. The analytical ability of the Deep5 pre-trained model with its segmented images for a detected pentagon and the generated velocity graphs with the detected peaks for shape analysis are then demonstrated. 

In Figure 4a where the original image is of two interlocking figures (left), both being pentagons, the segmented image (center) perceived by Deep5 has the estimated percentages $p_{f1}$ and $p_{f2}$ of 100.00% and 90.71%, respectively. The number of angles in each pentagon were both evaluated to be 5, as $p_{f1}$ and $p_{f2}$ were greater than the 0.75 score for $p_{th1}$, a threshold heuristically set by the two clinical experts in PDT scaling during the process of the ground truth scorings of all the images used in this study. For the velocity graph, the number of detected peaks corresponding to the number of angles in each figure was evaluated to be 5 (right). Figure 4b has the original drawing image of two interlocking figures, but only one is a pentagon (left figure). The segmented image by Deep5 has the estimated percentages $p_{f1}$ and $p_{f2}$ of 91.5% and 23.88%, respectively (center). The number of angles in the pentagon portion was evaluated to be 5, as the estimated percentage $p_{f1}$ was greater than 0.75; however, the number of angles in the non-pentagon portion was gauged to be 4, as the estimated percentage $p_{f2}$ was less than the 0.75 score and the number of peaks detected in the velocity graph was estimated to be 4 as well (right). Figure 4c similarly shows an example of an original drawing (left) of two interlocking figures with only one being a pentagon. The segmented image (middle) by Deep5 where the estimated percentages $p_{f1}$ and $p_{f2}$ were 29.10% and 98.52%, respectively. The number of angles in the non-pentagon portion was evaluated to be 4 from the estimated percentage score, $p_{f1}$, being less than 0.75. The number of peaks in the non-pentagon portion of the figure from the velocity graph (right) was estimated to be 4 as well. On the other hand, the number of angles of the pentagon portion was evaluated to be 5, as the estimated percentage score, $p_{f2}$, was greater than 0.75. Finally, the original drawing image (left) of Figure 4d depicts the example of two interlocking figures, with none of them being a pentagon. For the segmented image (middle) by Deep5, the estimated percentages, $p_{f1}$ and $p_{f2}$ were estimated to be 18.18 and 0.00%, respectively. In this case, the number of angles of both non-pentagons was evaluated to be 4, as both estimated percentages were less than 0.75 and the numbers of peaks detected in the velocity graph (right) were also estimated to be 4 for both non-pentagons.

### 3.2. Scoreing of Distance/Interlocking

**Figure 5:**
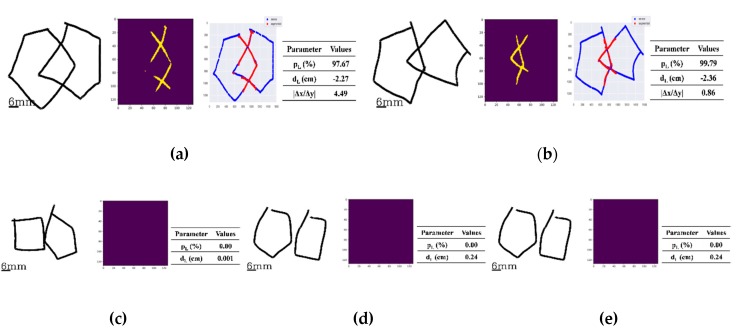
Five examples of the original drawing data along with the segmented images generated and analyzed by the pre-trained model, DeepLock (left and/or middle) and the corresponding parameter values (right). Below, the values in parentheses are the set comparator values for that parameter: (**a**) The case of the two interlocking figures giving a shape of a rhombus, where the percentage $p_{L}$, the distance $d_{L}$ and the absolute value |Δx/Δy| of the ratio were evaluated to be 97.67% ( > 0.75%), −2.27 cm ( < −0.01 cm) and 4.49 ( > 1.12), respectively. (**b**) The example of two interlocking figures, not giving a shape of a rhombus, where the percentage $p_{L}$, the distance $d_{L}$ and the absolute value |Δx/Δy| of the ratio were evaluated to be 99.79% ( > 0.75%), −2.36 cm (< −0.01 cm) and 0.86 ( < 1.12), respectively. (**c**) The case of two figures with no interlocking but still touching each other. There, the percentage $p_{L}$ and the distance $d_{L}$ were evaluated to be 0.00% ( < 0.75%) and 0.001 cm (being in between −0.01 and 0.01 cm range), respectively. (**d**) The display case of two figures, drawn so that they are separated from each other, but are within 1.00 cm of each other. The percentage $p_{L}$ and the distance $d_{L}$ were evaluated to be 0.00% ( < 0.75%) and 0.24 cm, respectively. (**e**) The example of two drawn figures that are more than 1.00 cm apart with the percentage $p_{L}$ and the distance $d_{L}$ were evaluated to be 0.00% (< 0.75%) and 1.50 cm, respectively.

The analytical ability of the pre-trained model DeepLock is demonstrated in Figure 5 with five separate examples of original drawing image along with the segmented image of interlocking generated by the program. Figure 5a has the original drawing image example (left) with the interlocking shape of a rhombus, its segmented image (middle) interlocking, and the overlap of the original drawing image along with the segmented image (right). In this case, the percentage $p_{L}$ and the distance $d_{L}$ were evaluated to be 97.67% and −2.27 cm, respectively. The absolute value |Δx/Δy| of the application was estimated to be 4.49. As such, the two figures were evaluated to be interlocked with a shape of a rhombus from three parameters: (1) percentage score, $p_{L}$, being greater than $p_{th2}$ of 0.75, a heuristically given threshold; (2) the absolute value |Δx/Δy| being larger than 1.12, a heuristically given threshold of $r_{th}$, and (3) the distance $d_{L}$ of a negative value equal to and less than 0.01 cm, as the threshold value of ε. Here, the threshold values for $p_{th2}$ and $r_{th}$ were chosen by the two clinical experts in PDT scaling during the process of the ground truth scorings of all the images used in this study. The threshold value ε was set to be 0.01 cm, considering a 2 pixel diagonal distance, 0.004 × 2 × sqrt(2), with a spatial resolution 0.004 cm of the device used in the implementation of the mPDT. The example in Figure 5b has the original drawing image of an interlocking shape that is not a rhombus (left) along with the resulting segmented image (middle) of the interlocking, and the overlap of the original drawing image and the segmented image (right). In this case, the percentage $p_{L}$ was evaluated to be 99.79% and the distance $d_{L}$ to be -2.36 cm. The absolute value |Δx/Δy| for the plot was estimated to be 0.86. From these parameters, the two figures were evaluated to be interlocked without a shape of a rhombus, having the percentage $p_{L}$ of greater than 0.75 for $p_{th2}$ and the distance *d_L_* of a negative value equal to and less than 0.01 cm for ε, but the absolute value |Δx/Δy| being less than 1.12 for $r_{th}$. 

In the example for Figure 5c, the two figures are not intersecting, but are making contact. The percentage $p_{L}$ was estimated to be 0.00% and the distance $d_{L}$ was calculated to be 0.001 cm, less than 0.01 cm for ε. In Figure 5d, the original image depicts two component figures that are apart from each other; the percentage $p_{L}$ was estimated to be 0.00% and the distance $d_{L}$ was calculated as to be 0.24 cm, greater than ε of 0.01 cm. The example in Figure 5e has two figures that are also apart from each other, giving the percentage $p_{L}$ estimated as 0.00% and the distance $d_{L}$ calculated as 1.50 cm.

### 3.3. Scoring of Closure/Opening

**Figure 6:**
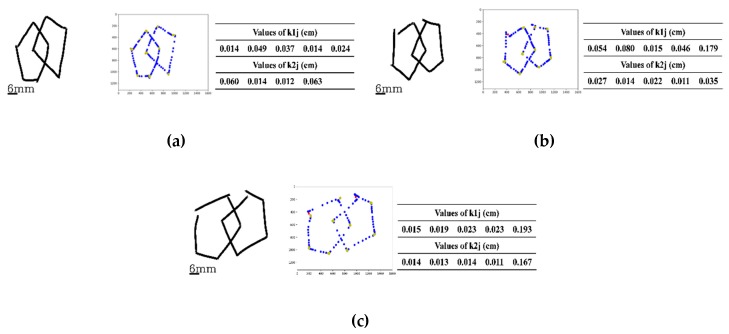
Three examples of openings being detected: (**a**) The case without any openings in both drawn figures. Here, the cluster parameters $k_{1j}$ and $k_{2j}$ of the left and right-portion figures were 0.014, 0.049, 0.037, 0.014, and 0.024 cm and 0.060, 0.014, 0.012, and 0.063 cm, respectively (all < 0.1 cm). (**b**) The example of a drawing with openings only in the left-portion of the figure. The cluster parameters $k_{1j}$ for the left-portion figure were 0.054, 0.080, 0.015, 0.046, and 0.179 cm (thus one >0.1cm); however, the cluster parameters $k_{2j}$ for the right-portion of the figure were 0.027, 0.014, 0.022, 0.011, and 0.035 cm (all < 0.1 cm). (**c**) The case of openings in both drawn figures with the cluster parameters $k_{1j}$ and $k_{2j}$ of the left and right figures of 0.015, 0.019, 0.023, 0.023, and 0.193 cm and 0.014, 0.013, 0.014, 0.011, and 0.167 cm, respectively (one for each figure > 0.1 cm).

In three representative examples, Figure 6 demonstrates how the openings in original images are detected and assigned. Figure 6a displays the case of an original image having no openings in the two interlockings. For the two interlocking figures, the cluster parameters $k_{1j}$ ($j=1,\ldots N_{A1}$) of the left interlocking figure were 0.014, 0.049, 0.037, 0.014, and 0.024 cm, respectively; for the right interlocking figure, $k_{2j}$ ($j=1,\ldots N_{A2}$) were 0.060, 0.014, 0.012, and 0.063 cm, respectively. Both sets of $k_{1j}$ and $k_{2j}$ values were less than the threshold $\delta_{th}$ of 0.1 cm for all their individual values. For Figure 6b, of the two interlocking figures, there is an opening for the left figure. $k_{1j}$ ($j=1,\ldots N_{A1}$) values for the left figure were 0.054, 0.080, 0.015, 0.046, and 0.179 cm, respectively, with one being greater than $\delta_{th}$ of 0.1 cm. For the right portion of the figure, the $k_{2j}$ ($j=1,\ldots N_{A2}$) values were 0.027, 0.014, 0.022, 0.011, and 0.035 cm, respectively, all of less than $\delta_{th}$ of 0.1 cm. The example in Figure 6c has an opening in each of the two interlocking figures. $k_{1j}$ ($j=1,\ldots N_{A1}$) for the left figure were 0.015, 0.019, 0.023, 0.023, and 0.193 cm, respectively, one of which was greater than $\delta_{th}$ of 0.1 cm. The $k_{2j}$ ($j=1,\ldots N_{A2}$) values for the right figure were 0.014, 0.013, 0.014, 0.011, and 0.167 cm, respectively, again with one being greater than $\delta_{th}$ of 0.1 cm, demonstrating an opening in the figure. Here, the threshold value $\delta_{th}$ was set to be 0.1 cm considering the line width (set to 20 pixels, ~0.08 cm) and the spatial resolution 0.004 cm of the device used in the implementation of the mPDT.

### 3.4. Scoring of Tremors

**Figure 7:**
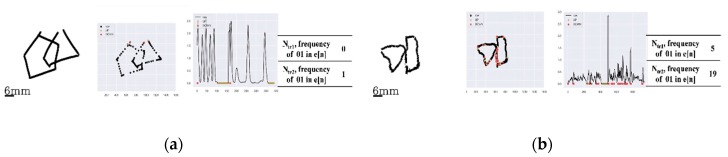
Two examples for detection of tremors: (**a**) The case without a detectable tremor, where the numbers of the ‘up’ events followed by ‘down’ events in the recorded touch-events were 0 and 1 for the left and right-portion figures, respectively (both < 5). (**b**) The case with detectable tremors where the numbers of the ‘up’ events followed by ‘down’ events in the recorded touch-events were 5 and 19 for the left and right-portion figures, respectively (one was >5).

For the presence of tremors in the hand drawn images, Figure 7 represents two cases with one having no tremors and the other having tremors. For Figure 7a with no significant tremors being present in either of the two interlocking figures, the total numbers of the ‘up’ events followed by ‘down’ events in touch-events e[n] of the sample points for the left and right portions of the interlocking figure were 0 and 1, respectively, and both with a given threshold $e_{th}$ of less than 5. The threshold value $e_{th}$ here was set by the two clinical experts in PDT scaling during the process of the ground truth scorings of all the images used in this study. In contrast, Figure 7b displays drawings with some tremors present in each of the two interlocking figures. In the figure, the ‘up’ events followed by ‘down’ events in the touch-events e[n] were 5 and 19 for the left and right figures, respectively, both being equal to or greater than 5, the given threshold $e_{th}$.

### 3.5. Performance Test Results

A total of 230 drawing images were used to test the performance of the scoring method with the mPDT. Table 4 summarizes the frequency of the ground truth for the 230 images with the score in each of the parameters. For the number of angles detected, the scores of 0 through 4 were 55, 32, 33, 30, and 80 events. For the distance/intersection parameter, the scores of 0 through 4 were for 33, 38, 34, 38, and 87 measures. Similarly, for the closure/opening measure, the scores of 0 through 2 were for 39, 61, and 130 detections, in the given order. The total numbers of instances for absence or presence of tremors, with a score of 0 or 1, were 16 and 214, respectively.

Table 5 lists the performance of each scoring parameter in mPDT. For the angle number parameter, the sensitivity, specificity, accuracy, and precision values were 97.53%, 92.62%, 94.35%, and 87.78%; for distance/intersection, they were 93.10%, 97.90%, 96.09%, and 96.43%; for closure/opening, they were 94.03%, 90.63%, 92.61%, and 93.33%; and for tremor reads, they were 100.00%, 100.00%, 100.00%, and 100.00%, respectively. 

## 4. Discussion

Conventional PDT based on a paper and pencil test is not readily suitable for evaluation of the dynamic components of cognitive function, as there are limitations in the real-time tracking of the orders, the stroke patterns, the speed variations, and so on, while the subjects are copying or drawing from recall. When subjects participate in a conventional PDT, many fMRI studies have shown multiple brain areas becoming active in the subject, including the bilateral parietal lobe, sensorimotor cortex, cerebellum, thalamus, premotor area, and inferior temporal sulcus [33,34,35,36]. However, it is not exactly clear what components of the cognitive function are associated with the activation of these areas as the conventional PDT is difficult to quantify objectively. To address this issue, our study focused on implementation of the PDT as a mobile phone application, namely mPDT, with a novel, automatic, and qualitative scoring method based on U-Net, a convolutional network for biomedical image segmentation of sensor data. The sensor data is also obtained by the mPDT. 

The performance test proved that the scoring protocol suggested by the mPDT is reasonable and practical when compared with those of the traditional PDT. Further, the mPDT was shown to be capable of evaluation of the dynamic components of cognitive function. In our study, the subjects used a smartpen provided for the smartphone when copying figures in order to create an environment similar to the conventional paper and pencil test of the PDT. This also increased the accuracy and avoided undesirable noise in the activated brain function assay. The performance test was restricted to right-handed subjects to avoid a bias in statistical analysis and also due to relatively small number of left-handed subjects available. However, the mPDT scoring worked quite in the same way when two left-handed subjects (a 27 year old male and a 26 year old female) were initially included in the younger volunteer group, both samples showing proper results and accuracies (see Appendix C).

The conventional PDT is a sub-item of MMSE, which is usually used in assessing Alzheimer’s disease [25]. However, the mPDT was developed to be applicable in better detection of cognitive impairment in Parkinson’s disease. For this reason, the tremor parameter was included in the scoring of the mPDT, instead of the closing-in parameter suggested in previous qualitative scoring of the pentagon test [5], and as the closing-in sign is a characteristic sign in all dementia, but not for Parkinson’s disease [37]. A tremor is an involuntary quivering movement or shake which may be due to age-associated weakness, a neurodegenerative disease, or a mental health condition. Therefore, there are several types of tremors recognized, such as essential tremors, Parkinsonian tremors, dystonic tremors, cerebellum tremors, psychogenic tremors, orthostatic tremors, and physiologic tremors [38]. In this study, the tremor symptom could be detected by the frequency of the occurrence of a ‘down’ event after an ‘up’ event in the touch-event series. A future study using mPDT for correlations between the pattern of tremoring and the underlying disease or condition could make a case for an early and differential diagnosis of a given neurodegenerative disease, such as Parkinson’s.

Conventional screening tools, including MMSE, do not detect early cognitive impairment in Parkinson’s disease, while the PDT is known to detect cognitive impairment earlier in Parkinson’s than in Alzheimer’s disease [39]. Using this fact, we aimed to develop a more sensitive screening tool to detect cognitive impairment in Parkinson’s. In addition, as the smartphone could evaluate the motor-related indicators such as the speed at which the test was performed or the number of pauses, we could also measure the effects on motor ability as a cognitive measurement tool that could not be detected by the conventional PDT pencil and paper test. Therefore, our study indicates that the developed mPDT tool is specifically applicable in increasing the accuracy of cognitive function assessment in Parkinson’s disease.

## 5. Conclusions

Even though the qualitative scorings of the PDT have been essential in establishing it as a prognostic marker in the assessment of visuospatial functions and in the differentiation of various neuronal degenerative diseases including Parkinson’s, the evaluation is done manually, which is not subjective, is prone to human error, and it is not able to provide parametric and dynamic information on a specific neuronal degenerative disease. In this study, we developed a smartphone application, named mPDT, with an automatic scoring method based on mobile sensors and image segmentation using the U-Net deep learning algorithm. A tremor read, not in the standard PDT, was also included, allowing for the detection of early Parkinson’s along with the other parameters tested. 

The mPDT is also relatively environment independent as it is applicable for different types of mobile devices, such as smartphones, tablets, and notebooks. It is also relatively fast. The execution time was 0.73 ± 0.21 seconds (mean ± std.) for the total score after a drawn PDT image was submitted in the performance test for a machine with Processor Intel(R) Core(TM) i7-8700 CPU at 3.20 GHz, 3192 Mhz 6 Core(s), 12 Logical Processor(s) with 8 GB of RAM running 64-bit Windows Version 10 specifications. 

The mPDT is very easy, simple and intuitive to use and it can be convenient for use by the elderly. Using mPDT for the PDT test also allows evaluation of the results objectively and qualitatively as well as for parametric assessment of the results. This can also allow differentiation of the dynamic components of the cognitive function underlying a given neurodegenerative disease. Furthermore, because redrawing and saving of the sensor data along with the images drawn by subjects are possible in any mobile, electronic device, the onset and time course of brain neuronal degeneration could be detected and monitored as a basis of a personal lifelog as well as in real time. Therefore, this tool is to evaluate the current cognitive functions of the examinee and better distinguish the causes of cognitive decline. 

For future work, we are currently developing qualitative and automatic scoring algorithms for the CDT and the ROCF tests by expanding the algorithms used in mPDT. Directions for this future work include various drawing tests, such as a draw-a-person test, a draw-a-family test, and so on, which would need more specific deep neural networks for image segmentation, feature extraction and classification, and clustering correlations between features. 

## Figures and Tables

**Table 1:** Statistics of age, gender, handedness, and clinical status of the participants. Pentagon drawing test (PDT).

	Training Set	Test Set	
	Volunteers (n = 199)	Volunteers (n = 129)	PD Patients (n = 101)
Age (mean ± standard deviation)	22.11 ± 1.44	26.87 ± 1.84	75.86 ± 8.13
Gender (male/female)	107/92	68/61	47/54
Binary PDT score (Pass/Nonpass )	199/0	48/81	32/69

**Table 2:** A detailed list of the parameters for the scoring method.

Parameters	Performance Scores	Assigned Integer Scores
**Number of Angles**	10	4
	9 or 11	3
	8 or 12	2
	7–5	1
	< 5 or > 13	0
**Distance/Intersection**	Correct intersection	4
	Wrong intersection	3
	Contact without intersection	2
	No contact, distance < 1 cm	1
	No contact, distance > 1 cm	0
**Closure/Opening**	Closure both figures	2
	Closure only one figure	1
	Opening both figures	0
**Tremor**	No tremor	1
	Tremor	0
**Total**		0–11

**Table 3:** Details for the assignment of scores.

Parameters	Assigned Integer Scores	Conditions (Scoring Method)
Number of angles	4	$p_{f1}\geq p_{th1}$ and $p_{f2}\geq p_{th1}$
	3	$p_{f1}<p_{th1}$ or $p_{f2}<p_{th1}$, $N_{A}{}_{1}+N_{A2}$ = 9 or 11
	2	$p_{f1}<p_{th1}$ or $p_{f2}<p_{th1}$, $N_{A}{}_{1}+N_{A2}$ = 8 or 12
	1	$p_{f1}<p_{th1}$ or $p_{f2}<p_{th1}$, 5 ≤ $N_{A}{}_{1}+N_{A2}$ ≤ 7
	0	$p_{f1}<p_{th1}$ or $p_{f2}<p_{th1}$, $N_{A}{}_{1}+N_{A2}$ < 5 or >13
Distance/intersection	4	$p_{L}\geq p_{th2}$, $d_{L}<-\varepsilon$, $\left|\Delta x/\Delta y\right|\geq r_{th}$
	3	$p_{L}<p_{th2}$, $d_{L}<-\varepsilon$, $\left|\Delta x/\Delta y\right|<r_{th}$
	2	$p_{L}<p_{th2}$, $\left|d_{L}\right|\leq \varepsilon$
	1	$p_{L}<p_{th2}$, $\varepsilon <d_{L}<1cm$
	0	$p_{L}<p_{th2}$, $d_{L}\geq 1cm$
Closure/opening	2	$N_{op1}^{}N_{op2}=1$, $N_{op1}^{}+N_{op2}=2$
	1	$N_{op1}^{}N_{op2}=0$, $N_{op1}^{}+N_{op2}=1$
	0	$N_{op1}^{}N_{op2}=0$, $N_{op1}^{}+N_{op2}=0$
Tremor	1	$N_{tr1}^{}N_{tr2}=1$
	0	$N_{tr1}^{}N_{tr2}=0$

**Table 4:** Frequency of the ground truth of the 230 images by score in each parameter of the scoring method of the mPDT.

Scores	Number of Angles	Distance/Intersection	Closure/Opening	Tremor
**4**	80	87	-	-
**3**	30	38	-	-
**2**	33	34	130	-
**1**	32	38	61	214
**0**	55	33	39	16
**total**	230	230	230	230

**Table 5:** Performance of the scoring parameters in the mPDT.

	Number of Angles	Distance/Intersection	Closure/Opening	Tremor
**TP**	79	81	126	214
**FP**	11	3	9	0
**FN**	2	6	8	0
**TN**	138	140	87	16
**Sensitivity**	97.53	93.10	94.03	100.00
**Specificity**	92.62	97.90	90.63	100.00
**Accuracy**	94.35	96.09	92.61	100.00
**Precision**	87.78	96.43	93.33	100.00

**Figure A1:**
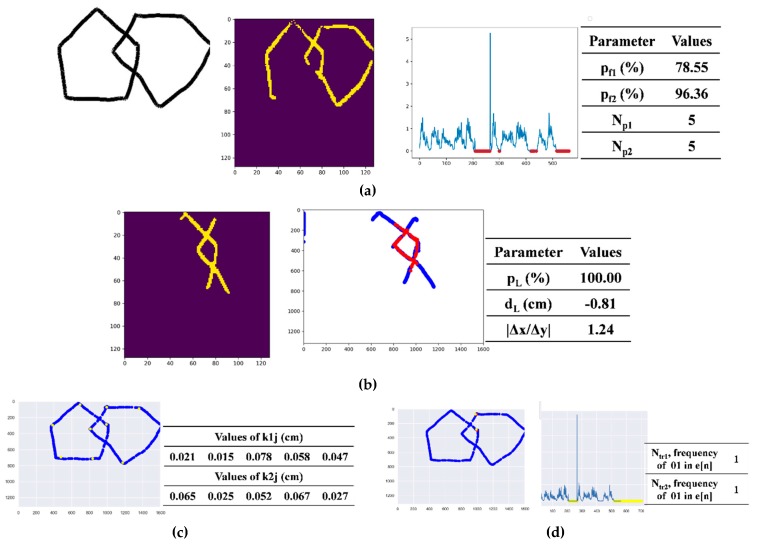
A case of a left-handed male subject (27 years old): (**a**) the original drawing (left) along with the segmented image (middle left) produced by the pre-trained model, Deep5, the corresponding velocity graph (middle right), the corresponding parameter values (right); (**b**) the segmented images generated and analyzed by the pre-trained model, DeepLock (left and middle) and the corresponding parameter values (right); (**c**) the clusters marked on the original drawing (left) and the corresponding parameter values (right); and (**d**) the up and down points marked on the original drawing (left) as well as on the velocity graph (middle) and the corresponding parameter values (right).

**Figure A2:**
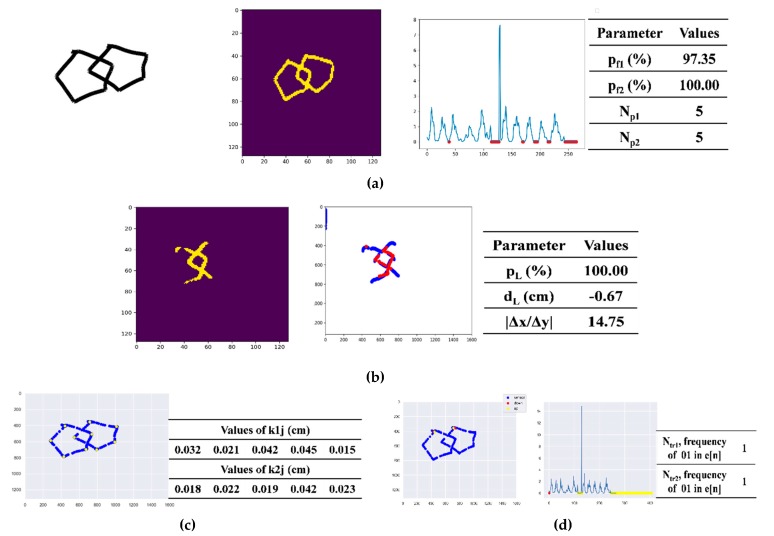
A case of a left-handed female subject (26 years old): (**a**) the original drawing (left) along with the segmented image (middle left) produced by the pre-trained model, Deep5, the corresponding velocity graph (middle right), the corresponding parameter values (right); (**b**) the segmented images generated and analyzed by the pre-trained model, DeepLock (left and middle) and the corresponding parameter values (right); (**c**) the clusters marked on the original drawing (left) and the corresponding parameter values (right); and (**d**) the up and down points marked on the original drawing (left) as well as on the velocity graph (middle) and the corresponding parameter values (right).

## Appendix A

**Table A1:** Summary and comparison of the present study and the existing literature.

Mode of Drawing	Test Type	Scoring Method and Spec.	Reference
**Clock drawing test (CDT)**	paper-based	quantitative, semi-quantitative 5, 6, 10, 12, 20 points systems manually interpreting	[16] 2018 review
	digital CDT	semi-quantitative automatic estimation of stroke features, up and down 6 points system manually interpreting based on computerized feature	[17] 2017
	digital CDT	qualitative ontology-based knowledge representation CNN for object recognition automatically recognize each number by the probability score	[18] 2017
	digital CDT	qualitative categorized stroke data based on feature using machine learning	[19] 2016
	digital CDT	qualitative automatic estimation of stroke features, up and down, stroke velocity, stroke pressure	[20] 2019
**Rey–Osterrieth complex figure (ROCF)**	paper-based	quantitative location and perceptual grading of the basic geometric features	[21] 1944
	paper-based	qualitative points 0-24 based on the order in which the figure is produced	[23] 2017
	paper-based	quantitative automated scoring of 18 segments based on a cascade of deep neural networks trained on human rater scores	[24] 2019
**PDT**	paper-based	qualitative 6 points system manually interpreting	[25] 1995
	paper-based	qualitative 10 points system manually interpreting	[26] 2011
	paper-based	qualitative 6 sub scales manually interpreting based on factor analysis and 6 subscales correlated to control responses	[27] 2012
	paper-based	qualitative points 0–13 based on parametric estimations for number of angles, distance/intersection, closure/opening, rotation, closing-in manually interpreting	[5] 2013
**mPDT**	mobile-based	qualitative points 0–11 based on stroke feature for the order and the speed and also parametric estimations for number of angles, distance/intersection, closure/opening, tremor automatic interpreting using U-net deep learning algorithm and sensor data	The presented system

## Appendix B

**Table A2:** Examples of corresponding drawings and scores for each level with the corresponding detailed parameters.

Case Image	Performance Scores				Assigned Integer Scores				Total Score
	NA^1^	D/I^2^	C/O^3^	Tr^4^	NA^1^	D/I^2^	C/O^3^	Tr^4^	
	**10**	**CI^5^**	**NO^10^**	**NT^13^**	**4**	**4**	**2**	**1**	**11**
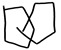
	**10**	**CI^5^**	**O1^11^**	**NT^13^**	**4**	**4**	**1**	**1**	**10**
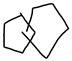
	**10**	**WI^6^**	**NO^10^**	**NT^13^**	**4**	**3**	**2**	**1**	**10**
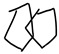
	**10**	**CI^5^**	**O2^12^**	**NT^13^**	**4**	**4**	**0**	**1**	**9**
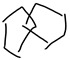
	**10**	**WI^6^**	**O1^11^**	**NT^13^**	**4**	**3**	**1**	**1**	**9**
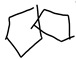
	**10**	**NC0^8^**	**NO^10^**	**NT^13^**	**4**	**1**	**2**	**1**	**8**
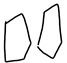
	**10**	**C^7^**	**O1^11^**	**NT^13^**	**4**	**2**	**1**	**1**	**8**
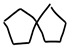
	**9**	**CI^5^**	**NO^10^**	**NT^13^**	**3**	**4**	**2**	**1**	**10**
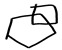
	**9**	**CI^5^**	**O1^11^**	**NT^13^**	**3**	**4**	**1**	**1**	**9**
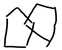
	**9**	**WI^6^**	**NO^10^**	**NT^13^**	**3**	**3**	**2**	**1**	**9**
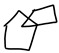
	**9**	**C^7^**	**NO^10^**	**NT^13^**	**3**	**2**	**2**	**1**	**8**
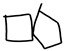
	**9**	**WI^6^**	**O1^11^**	**NT^13^**	**3**	**3**	**1**	**1**	**8**
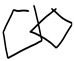
	**9**	**WI^6^**	**O2^12^**	**NT^13^**	**3**	**3**	**0**	**1**	**7**
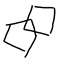
	**9**	**C^7^**	**O1^11^**	**NT^13^**	**3**	**2**	**1**	**1**	**7**
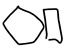
	**11**	**NC0^8^**	**O2^12^**	**NT^13^**	**3**	**1**	**0**	**1**	**5**
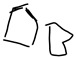
	**8**	**CI^5^**	**NO^10^**	**NT^13^**	**2**	**4**	**2**	**1**	**9**
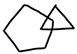
	**8**	**CI^5^**	**NO^10^**	**NT^13^**	**2**	**4**	**2**	**1**	**9**
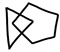
	**8**	**WI^6^**	**NO^10^**	**NT^13^**	**2**	**3**	**2**	**1**	**8**
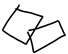
	**8**	**WI^6^**	**NO^10^**	**NT^13^**	**2**	**3**	**2**	**1**	**8**
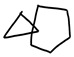
	**8**	**WI^6^**	**O1^11^**	**NT^13^**	**2**	**3**	**1**	**1**	**7**
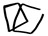
	**8**	**C^7^**	**NO^10^**	**NT^13^**	**2**	**2**	**2**	**1**	**7**
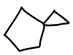
	**8**	**NC0^8^**	**NO^10^**	**NT^13^**	**2**	**1**	**2**	**1**	**6**
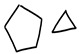
	**8**	**NC0^8^**	**O1^11^**	**NT^13^**	**2**	**1**	**1**	**1**	**5**
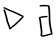
	**8**	**NC1^9^**	**NO^10^**	**NT^13^**	**2**	**0**	**2**	**1**	**5**
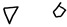
	**8**	**NC0^8^**	**O2^12^**	**NT^13^**	**2**	**1**	**0**	**1**	**4**
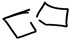
	**7**	**CI^5^**	**NO^10^**	**NT^13^**	**1**	**4**	**2**	**1**	**8**
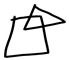
	**7**	**NC0^8^**	**NO^10^**	**NT^13^**	**1**	**1**	**2**	**1**	**5**
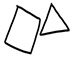
	**7**	**NC1^9^**	**NO^10^**	**NT^13^**	**1**	**0**	**2**	**1**	**4**
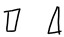
	**7**	**NC1^9^**	**O1^11^**	**NT^13^**	**1**	**0**	**1**	**1**	**3**
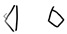
	**6**	**NC1^9^**	**O1^11^**	**NT^13^**	**1**	**0**	**1**	**1**	**3**
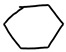
	**>13**	**WI^6^**	**NO^10^**	**NT^13^**	**0**	**3**	**2**	**1**	**6**
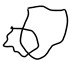
	**>13**	**CI^5^**	**O2^12^**	**NT^13^**	**0**	**4**	**0**	**1**	**5**
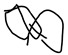
	**>13**	**NC0^8^**	**O1^11^**	**NT^13^**	**0**	**1**	**1**	**0**	**2**
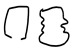
	**>13**	**C^7^**	**O2^12^**	**T^14^**	**0**	**2**	**0**	**0**	**2**
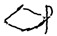
	**4**	**NC1^9^**	**O2^12^**	**NT^13^**	**0**	**0**	**0**	**1**	**1**
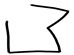
	**4**	**NC1^9^**	**O2^12^**	**T^14^**	**0**	**0**	**0**	**0**	**0**
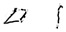

^1^NA: Number of angles; ^2^D/I: Distance/interaction; ^3^C/O: Closure/opening; ^4^Tr: Tremor. ^5^CI: Correct intersection; ^6^WI: Wrong intersection; ^7^C: Contact without intersection; ^8^NC0: No contact, distance<1cm; ^9^NC1: No contact, distance>1cm; ^10^NO: Closure both figures; ^11^O1: Closure only one figure; ^12^O2: Opening both figures; ^13^NT: No Tremor; ^14^T: Tremors.

## Appendix C

**Table A3:** The corresponding drawings and scores for each level with the corresponding detailed parameters of the case of two left-handed subjects.

Case of Image of the Left-Handed Subject	Performance Scores				Assigned Integer Scores				Total Score
	NA^1^	D/I^2^	C/O^3^	Tr^4^	NA^1^	D/I^2^	C/O^3^	Tr^4^	
	**10**	**CI^5^**	**NO^6^**	**NT^7^**	**4**	**4**	**2**	**1**	**11**
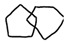
	**10**	**CI^5^**	**NO^6^**	**NT^7^**	**4**	**4**	**2**	**1**	**11**
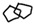

^1^NA: Number of angles; ^2^D/I: Distance/interaction; ^3^C/O: Closure/opening; ^4^Tr: Tremor; ^5^CI: Correct intersection; ^6^NO: Closure both figures; ^7^NT: No Tremors.
